# 5-alpha-reductase type I (SRD5A1) is up-regulated in non-small cell lung cancer but does not impact proliferation, cell cycle distribution or apoptosis

**DOI:** 10.1186/1475-2867-12-1

**Published:** 2012-01-18

**Authors:** Friedrich G Kapp, Anette Sommer, Thomas Kiefer, Gottfried Dölken, Bernard Haendler

**Affiliations:** 1 Global Drug Discovery, Bayer HealthCare, Müllerstr. 178, 13342 Berlin, Germany; 2 Internal Medicine C (Haematology and Oncology, Transplant Centre), Ernst-Moritz-Arndt-University, Greifswald, Germany

## Abstract

**Background:**

Non-small cell lung cancer (NSCLC) is one of the most frequent malignancies and has a high mortality rate due to late detection and lack of efficient treatments. Identifying novel drug targets for this indication may open the way for new treatment strategies. Comparison of gene expression profiles of NSCLC and normal adjacent tissue (NAT) allowed to determine that 5-alpha-reductase type I (SRD5A1) was up-regulated in NSCLC compared to NAT. This raised the question whether SRD5A1 was involved in sustained proliferation and survival of NSCLC.

**Methods:**

siRNA-mediated silencing of SRD5A1 was performed in A549 and NCI-H460 lung cancer cell lines in order to determine the impact on proliferation, on distribution during the different phases of the cell cycle, and on apoptosis/necrosis. In addition, lung cancer cell lines were treated with 4-azasteroids, which specifically inhibit SRD5A1 activity, and the effects on proliferation were measured. Statistical analyses using ANOVA and post-hoc Tamhane-T2-test were performed. In the case of non-parametric data, the Kruskal-Wallis test and the post-hoc Mann-Whitney-U-test were used.

**Results:**

The knock-down of SRDA51 expression was very efficient with the SRD5A1 transcripts being reduced to 10% of control levels. Knock-down efficiency was furthermore confirmed at the protein level. However, no effect of SRD5A1 silencing was observed in the proliferation assay, the cell cycle analysis, and the apoptosis/necrosis assay. Treatment of lung cancer cell lines with 4-azasteroids did not significantly inhibit proliferation.

**Conclusions:**

In summary, the results suggest that SRD5A1 is not a crucial enzyme for the sustained proliferation of NSCLC cell lines.

## Background

Lung cancer remains one of the leading causes of morbidity and mortality in cancer patients, and its prognosis is unsatisfactory with an overall survival of 15-18% [1]. Surgical resection promotes good long-term survival in the early stages with up to 57%-71% in stage I non-small cell lung cancer (NSCLC) [2] and 33-57% in stage II NSCLC [3]. Unfortunately, late diagnosis is common due to the lack of early symptoms, which explains the low survival rates. Chemotherapy in the late stages III and IV did improve the outcome in the last decades, but only slightly from 10% in the 1980s to 15-18% nowadays. Thus, new and more effective treatment strategies are urgently needed. Several genome-wide gene expression profiling studies have been performed in the past years (reviewed by Mueller-Hagen et al. [4]). These analyses provide valuable diagnostic and prognostic markers as well as a basis for the discovery of novel target candidates for the therapy of NSCLC. More recently, a genome-wide expression profiling analysis of matching pairs of NSCLC and normal adjacent tissue (NAT) on Affymetrix exon arrays was performed, providing evidence for genes alternatively spliced in NSCLC [5].

Two main steroid 5-alpha-reductases have been identified, 5-alpha-reductase type I (SRD5A1) and 5-alpha-reductase type II (SRD5A2) [6]. The recently described type III (SRD5A3) [7] was originally identified in prostate cancer tissue and acts as a polyprenol reductase involved in the *N*-linked glycosylation of proteins [8]. SRD5A1 is expressed mainly in the skin from the time of puberty while SRD5A2 is the predominant enzyme in the prostate and male accessory sex glands [9]. While SRD5A2 is an initiating factor for male pattern baldness, its germ line deficiency results in male pseudohermaphroditism, which is characterized by phenotypically female external genitalia at birth. It has been suggested that SRD5A1 and SRD5A2 are involved in the pathogenesis of polycystic ovary syndrome [10]. So far, only few publications examined the role of 5-alpha-reductases in the lung and in lung cell lines, with partly conflicting results. Provost et al. detected only a small amount of SRD5A1 mRNA and only little enzymatic activity of SRD5A1 in A549 lung cancer cells [11]. In a microarray analysis of different tissues, SRD5A1 expression has been described as being low in normal lung tissue [12]. So far, there have been no reports on the expression level of SRD5A1 mRNA in NCI-H460 cells. Over-expression of SRD5A1 and SRD5A2 has been noted in breast and prostate cancer samples [13,14].

In preliminary studies with Affymetrix GeneChips, SRD5A1 was identified as up-regulated in NSCLC compared to NAT. The hypothesis was developed that SRD5A1 was possibly involved in sustaining the proliferation in NSCLC cell lines. In order to analyze this, knock-down of SRD5A1 expression was performed and the effects on cell growth, cell cycle distribution, apoptosis, and necrosis were determined. In addition, the impact of blocking the enzymatic activity of SRD5A1 with compounds selective for SRD5A1 such as 4-azasteroids was studied.

## Methods

### Clinical samples

Lung cancer samples and NAT were obtained with informed consent from patients treated at the Department of General, Vascular and Thoracic Surgery and at the Institute of Pathology, Charité, Universitätsmedizin Berlin, Campus Benjamin Franklin, Berlin, Germany [5].

### Cell lines and culture

The human NSCLC cell lines A549 and NCI-H460 (H460) were obtained from ATCC (ATCC, Manassas, VA, USA). Both cell lines were cultured in DMEM/Ham's F12 medium (Biochrom AG, Berlin, Germany) supplemented with 2 mM L-glutamine (Invitrogen, Carlsbad, CA, USA), 100 units/ml penicillin G and 100 μg/ml streptomycin (Invitrogen). Also, fetal calf serum (FCS; Biochrom AG), which contains 0.03 ng/ml testosterone, was added to a final concentration of 10%. The cells were grown at 37.0°C, in 5% CO_2 _and 95% relative humidity.

### Affymetrix microarray analysis

RNA was transcribed into cRNA and hybridized onto Affymetrix GeneChip HG-U133Plus2.0 The hybridization intensities on each array were calculated with the MAS5.0 summarization algorithm. The refined and summarized data were loaded into the CoBi database (Genedata, Basel, CH). The analysis of the probeset-specific signal intensities was performed with the Genedata Expressionist Version 6.1 software (Genedata, Basel, CH). The dataset was normalized using Central Tendency Median Normalization. The Genedata software was used for Principal Component Analysis (PCA) and statistical tests. SRD5A1 expression was analyzed using the in-house available Array Northern database [15]. Here, the expression values for a specific gene were displayed as a bar graph of the geometric mean values of the expression value on an arbitrary scale over all samples belonging to a specific class (e.g. tissue or organ type, cell line). For a number of organs, the expression levels in corresponding cancer samples are additionally shown. First, the expression data were transformed into logarithmic scale, then a calculation of the arithmetic mean and standard deviation was performed. Afterwards, the mean values were transformed to mean +/- standard deviation on a linear scale.

### siRNA knock-down studies

Stealth™ siRNAs (Invitrogen) were used for the knock-down experiments. In total, three different siRNAs targeting SRD5A1 and three mismatch siRNAs were used (see Table 1 for details). The siRNAs were screened for sequence similarities with NCBI BLAST using the "Human genomic plus transcript" database in the nucleotide BLAST setting. All three siRNAs directed against human SRD5A1 were 100% identical to the transcript of human SRD5A1 with a query coverage of 100% and an E-value of 4 × 10^-5 ^(see also Additional File 1). Few other transcripts occurred in the BLAST and they showed only little similarity (52-60% query coverage, E-value of 33-509) and were considered irrelevant. Mismatch siRNAs were also blasted and showed virtually no similarity to any transcript (52-56% query coverage, E-value of 129-509). Unwanted off-target effects were therefore not expected for the selected siRNAs. Results are displayed for transcript matches only. The binding sites of the target siRNAs (siRNA 1-3) are displayed in Additional File 1.

**Table 1:** Human SRD5A1 specific and mismatch (mm) siRNAs.

Abbreviation	Target	Name	Sequence
siRNA 1	SRD5A1	NM_001047.1_STEALTH_406	CCACTACGGGCATCGGTGCTTAATT

mm 1	Mismatch	NM_001047.1_STEALTH_CTL_406	CCAGGCATACGTGGCTTCGATCATT

siRNA 2	SRD5A1	NM_001047.1_STEALTH_ 542	GCAGTGTATGCTGATGACTGGGTAA

mm 2	Mismatch	NM_001047.1_STEALTH_ CTL_542	GCATATGGTCGAGTAGGTCGTCTAA

siRNA 3	SRD5A1	NM_001047.1_STEALTH_701	GAATACGTAACTGCAGCCAACTATT

mm 3	Mismatch	NM_001047.1_STEALTH_ CTL_701	GAAATGCGTCACGACCAACTATATT

### Transfection

Lipofectamine™ 2000 (Invitrogen) and OPTI-MEM I (Invitrogen) were used for the transfection of siRNA into cultured cells. BLOCK-iT™ Fluorescent Oligo (Invitrogen) was used to visualize successful transfection via fluorescence microscopy. The lowest amount of siRNA required for a consistent knock-down of SRD5A1 was determined to be 10 pmol. For transfection, 5 μl of Lipofectamine™ 2000 were used per well of a six-well culture plate (TPP, Trasadingen, Switzerland). In order to reduce possible toxic effects, the amount of Lipofectamine™ 2000 was lowered to 2.5 μl per well in the course of the experiments.

### Expression analysis by quantitative real-time PCR (qRT-PCR)

RNA was extracted with the RNeasy^® ^Mini Kit (Qiagen, Hilden, Germany), followed by digestion of genomic DNA using the RNase free DNase-Set (Qiagen). RNA content was measured by spectrophotometry. The cDNA synthesis was conducted with SuperScript™ III First-Strand Synthesis System for RT-PCR (Invitrogen) using 1 μg of the extracted total RNA. The cDNA was analyzed in a multiplex analysis using TaqMan^® ^Universal PCR MasterMix and specific primers for SRD5A1 or the internal controls (Applied Biosystems, Foster City, CA, USA). The primers specific for SRD5A1 had the Assay ID: Hs00602694_mH, reporter dye FAM). Cyclophilin A served as the endogenous control and had the reference 4310883E (VIC/TAMRA Probe, Primer Limited). The reagents were assembled in MicroAmp™ Fast Optical 96-Well Reaction Plates (Applied Biosystems) and the PCR reaction was conducted in a 7500 Fast Real-Time PCR System (Applied Biosystems). The regular RT-PCR program was used and the read-out was set to automatic threshold (C_t_). Each cell culture sample was measured in triplicates, non-template controls were included to detect potential contamination. In order to apply the ΔΔC_t _method, a validation experiment according to the protocol provided by Applied Biosystems [16] was conducted first, to demonstrate that multiplex analysis of SRD5A1 and cyclophilin was feasible. The validation experiment was successful with an absolute value of the slope of ΔC_t _vs. log input of RNA being < 0.1 (-0,07, data not shown). The qRT-PCR protocol from Applied Biosystems [15] and the publication by Yuan et al [17] describe the ΔΔC_t_-method used for analysis of the real-time PCR data. In brief, the cycle number at the C_t _for the target gene (SRD5A1) was subtracted by the C_t _of the reference gene (cyclophilin A) to give the ΔC_t _value. The ΔC_t _of the treated sample was then subtracted from the ΔC_t _of the untreated sample to give ΔΔC_t_. The exponential function of 2^-ΔΔC^t represents the relative amount of the target gene in the treated sample compared to the target gene in the untreated control.

For the quantitative expression analysis of lung carcinoma and NAT samples obtained from patients, additional SRD5A1 primers were used (Assay ID Hs00971643_g1, reporter Dye FAM), as well as a second internal control, namely 18S rRNA (Reference 4319413E, VIC/MGB probe, Primer Limited). RNA and cDNA were prepared as before, and the regular RT-PCR program used to determine C_t _values. Each sample value was obtained by the average of duplicate experiments. The mean C_t _value (raw means and standard deviation) measured for the different primers is shown in Table 2. For statistical analysis, the repeated measures design (per patient: lung carcinoma and NAT, SRD5A1 and control primer pairs) was taken into account and a mixed linear model was used for fitting.

**Table 2:** Expression levels of SRD5A1 in NSCLC and NAT samples as measured by qRT-PCR.

Primer target	Analyzed biopsy	Number of samples	Average C_t_	Average standard deviation
SRD5A1 #1	NAT	9	30.74	0.97
	
	tumor	23	29.25	1.37

SRD5A1 #2	NAT	9	31.30	1.16
	
	tumor	23	29.45	1.69

Cyclophilin	NAT	9	21.28	0.52
	
	tumor	23	20.46	0.98

18S rRNA	NAT	9	16.94	0.56
	
	tumor	23	17.01	0.67

The analysis was performed on tumor samples from 23 patients, of which 9 provided NAT as well.

### Immunoblot

The cells were suspended in M-PER buffer (Pierce Biotechnology, Rockford, IL, USA) for protein extraction. The protein lysate was then run on NuPAGE 12% Bis-Tris Gel (Invitrogen) and blotted onto PVDF membranes (Invitrogen). The membranes were then rinsed with 0.1% Tween^® ^20 (Carl Roth GmbH + Co. KG, Karlsruhe, Germany) and blocked with 5% milk powder (Carl Roth GmbH). Rabbit polyclonal anti-5-alpha-reductase 1 H-105 (Santa Cruz Biotechnology, Inc., Santa Cruz, CA, USA) and goat anti-SRD5A1 / 5-alpha-reductase antibodies (Everest Biotech Ltd., Upper Heyford, UK) were used with the respective secondary antibodies for detection of SRD5A1. Mouse anti-GAPDH antibody (Advanced Immunochemical, Long Beach, CA, USA) was used as loading control. The two anti-SRD5A1 antibodies either resulted in no bands detected on the immunoblot or multiple non-specific bands and could not be used for determination of SRD5A1 protein levels after knock-down. A plasmid containing a cDNA of the human SRD5A1 gene with a V5-epitope at the C-terminus was therefore used to overexpress SRD5A1 in the two lung cancer cell lines and a mouse monoclonal anti-V5 antibody (Invitrogen) was used for detection. A sheep ECL Anti-Mouse IgG, Horseradish Peroxidase-Linked Antibody (Amersham Biosciences, GE Healthcare Bio-Sciences AB, Uppsala, Sweden) was used as a secondary antibody. The immunoblot was developed with Western Lightning Reagent (PerkinElmer Inc., Waltham, MA, USA) on Amersham Hyperfilm ECL (GE Healthcare Life Sciences, Munich, Germany) in a Curix 60 device (Agfa, Mortsel, Belgium). The immunoblots were analyzed with the ImageJ software (by Waynde Rasband (NIH), Washington D.C., USA) according to the protocol by Miller [18].

### Plasmid transformation, purification and transfection

Due to the difficulties in quantifying the reduction of SRD5A1 protein with commercially available SRD5A1 antibodies, an immunoblot with antibodies against the V5 epitope was performed. The expression construct was generated by cloning human SRD5A1 cDNA into the pcDNA/V5-His vector (Invitrogen), for details see Additional File 2. Plasmid transformation was conducted with XL1-Blue Supercompetent Cells (Stratagene, Santa Clara, CA, USA) and the heat-shock method at 42°C for 45 s. Cells were then cooled and incubated on agar plates at 37°C overnight. Single colonies were selected and transferred into LB-medium containing ampicillin and shaken at 37° overnight. The cells were then centrifuged and processed with the QIAfilter Plasmid Kit (QIAGEN) to extract recombinant plasmid. Purified plasmid (0.5 μg) was then transfected into A549 and NCI-H460 cells using 5 μl of Lipofectamine™ 2000. In order to verify successful transfection, a GFP-containing plasmid was transfected in a separate well and fluorescence microscopy was conducted the next day. After 24 h, the cells were transfected with siRNAs following the method described above using 10 μl siRNA and 3.75 μl Lipofectamine™ 2000. The cells were harvested 24 h and 48 h after siRNA transfection.

### Proliferation assay

24 h, 48 h and 72 h after transfection of siRNAs, AlamarBlue (BioSource Europe, Nivelles, Belgium) was added to the cultured cells which were then incubated for 1 h 45 min before being measured in a Victor3 1420 Multilabel Counter (Perkin Elmer Inc.) (excitation 530 nm, emission 590 nm). The results were compared to the 24 h time-point of non-treated control cells and are displayed as percentage.

### Cell cycle analysis

24 h, 48 h and 72 h after transfection with siRNAs, cells were collected, washed twice with PBS, fixed in 70% ethanol and frozen overnight. After thawing, RNase A (Sigma-Aldrich, St. Louis, MO, USA) and propidium iodide (Sigma-Aldrich) were added. Distribution in the cell cycle was analyzed on a FACSCalibur (BD Biosciences, San Jose, CA, USA) flow cytometer using the CellQuest Pro software (BD Biosciences). A total of 10,000 cells was counted per treatment group.

### Apoptosis/necrosis assay

24 h, 48 h and 72 h after transfection with siRNAs, cells were collected and washed with PBS. The Annexin V-FITC Apoptosis Detection Kit I (BD Biosciences) was used for detection of apoptosis and necrosis. In brief, the cells were washed with PBS and afterwards with Annexin Binding Buffer. After centrifugation, the supernatant was discarded, Binding Buffer as well as Annexin V-FITC and propidium iodide were added to the cells which were incubated for 15 min at room temperature in the dark. After incubation, Binding Buffer was added and the analyses were performed in the flow cytometer mentioned above, with 10,000 cells being counted per treatment group.

### Chemosensitivity assay

Three 17-methylene-4-azasteroids (see Additional File 3) inhibiting SRD5A1 selectively [19] (ZK-879, ZK-924, ZK-425 [20]; Jenapharm GmbH, Jena, Germany) and Finasteride (Sigma), a SRD5A2 inhibitor, were diluted in ethanol to concentrations ranging from 10^-5 ^M to 10^-9 ^M. The fully synthetic sagopilone (ZK-EPO) (Bayer HealthCare, Berlin, Germany), a microtubule inhibitor [21],[22] was used as positive control for proliferation inhibition at concentrations ranging from 10^-7 ^M to 10^-9 ^M. The viability of the cells was assessed in the proliferation assay described above at 24 h, 72 h, and 120 h. Ethanol diluted at the appropriate concentration was used as vehicle control.

### Statistical analyses of cell culture experiments

For parametrical data, a two-tailed one-factor ANOVA was conducted, with Dunnett's test in case of equal variances and Tamhane's T2 in case of unequal variances. If the data were non-parametric, a Kruskal-Wallis-Test was performed, the post-hoc analysis was done with a Mann-Whitney-U-Test. The threshold for significant results was p < 0.05. Significant results are indicated in the legends of the figures. All experiments except flow cytometry were conducted three times, each one with three replicates. Flow cytometry analyses were conducted three times, each one with one sample (10,000 analyzed cells), making the calculation of a p-value not useful but giving reliable results based on approximately 30,000 analyzed cells.

## Results

### Expression analysis of SRD5A1 in lung tumor samples and cell lines

**Figure 1:**
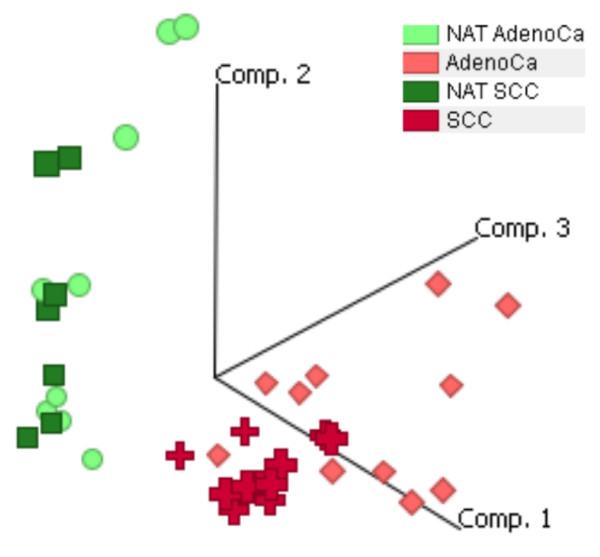
**PCA allows to distinguish NSCLC from NAT**. Ten cases of adenocarcinomas (AdenoCa) and nine NAT matching samples, and eleven cases of SCC and seven NAT matching samples were hybridized to Affymetrix HGU133Plus2.0 arrays. All NSCLC samples (indicated in shades of red) clustered separately from the NAT samples (indicated in shades of green).

A total of 37 patients' samples, ten cases of adenocarcinomas and nine matching samples of NAT from adenocarcinoma (AdenoCa), eleven cases of squamous cell carcinoma (SCC) and seven matching samples of NAT from SCC, were obtained. RNA was isolated, labeled and hybridized to Affymetrix HGU133Plus2.0 arrays. The results were analyzed with the Expressionist software. Following normalization, a PCA was performed (Figure 1). The PCA demonstrated that all adenocarcinoma and SCC samples clustered separately from all NAT samples. Two different two-group tests were applied for comparison of NSCLC and NAT, the Welsh test and the N-fold regulation test. The identified gene groups were merged and K-means clustering was performed. Groups of up- and down-regulated genes were identified. Several criteria such as consistency of over-expression, tissue distribution, potential role in a disease process, as well as druggability were then used to select the most interesting target candidate genes for validation [15].

**Figure 2:**
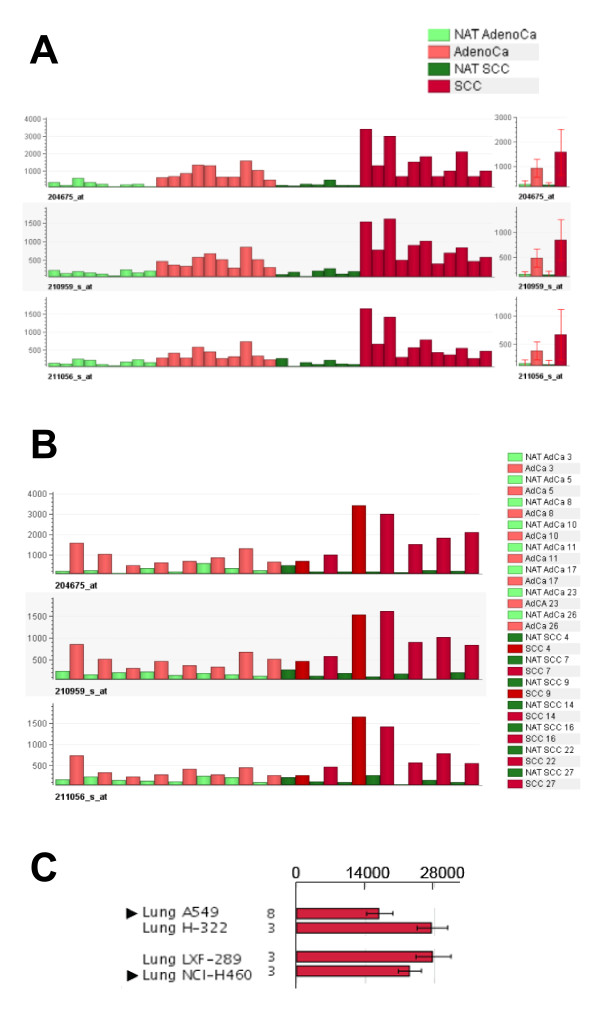
**Expression of SRD5A1 in lung tumors, NAT and lung cell lines**. (A) Three independent probesets (204675_at, 210959_at, and 211056_at) were used to measure the SRD5A1 mRNA levels using the HGU133Plus2.0 array. The bar chart indicates that SRD5A1 was significantly and consistently up-regulated in the NSCLC samples compared to NAT. Comparison of the signal intensities showed a 3.3- and 6.3-fold stronger signal of SRD5A1 mRNA in adenocarcinoma and SCC of the lung, respectively, when compared to NAT. The mean values with one standard deviation are depicted on the right. (B) For eight cases of adenocarcinoma (AdenoCa) and seven cases of SCC, matching samples from cancer and NAT were available. Here, a pairwise comparison of the samples is displayed. In all paired cases, SRD5A1 is expressed at a higher level in the cancer than in the NAT samples. (C) Four NSCLC cell lines were analyzed using the Array Northern database. The results for the SRD5A1-specific probeset 204675_at are shown. The numbers indicate the number of replicates analyzed. Expression levels in the NSCLC cell lines A549 and NCI-H460 (indicated with an arrowhead), and LXF-289 and NCI-H322 (abbreviated H-322) are shown.

It was found that the expression of SRD5A1 was significantly up-regulated in the examined NSCLC samples compared to NAT. Comparison of the signal intensities showed a 3.3- and 6.3-fold stronger signal of SRD5A1 mRNA in adenocarcinoma and SCC of the lung, respectively, when compared to NAT (Figure 2A). For nine cases of adenocarcinoma and seven cases of cases of SCC, matching samples from cancer and NAT were available. Here, a pairwise comparison of the samples is displayed. In all paired cases, SRD5A1 was expressed at a higher level in the cancer than in the NAT samples (Figure 2B).

In order to further substantiate these data, clinical samples from 23 additional lung cancer patients were analyzed. One tumor sample was obtained from each patient and in 9 cases an NAT sample was available as well. Following RNA extraction and cDNA synthesis, qRT-PCR was performed using two different primer sets specific for SRD5A1. In parallel, the expression levels for the housekeeping gene cyclophilin A and for 18S rRNA were determined. Comparison of the measured C_t _values showed that for these samples also, there was a significantly higher expression of SRD5A1 in lung tumors than in NAT (p = 0.0007 for SRD5A1 primer pair #1, p = 0.0011 for SRD5A1 primer pair #2; Table 2). Conversely, the levels of cyclophilin A and of 18S rRNA did not differ significantly between both groups (p = 0.3008 and p = 0.6570, respectively; Table 2).

Finally we determined the expression of SRD5A1 in cell lines originating from NSCLC. Similar transcript levels were measured in A549, NCI-H322, NCI-H460 and LXF-289 cells (Figure 2C).

### Expression analysis of SRD5A1 in other tumors

**Figure 3:**
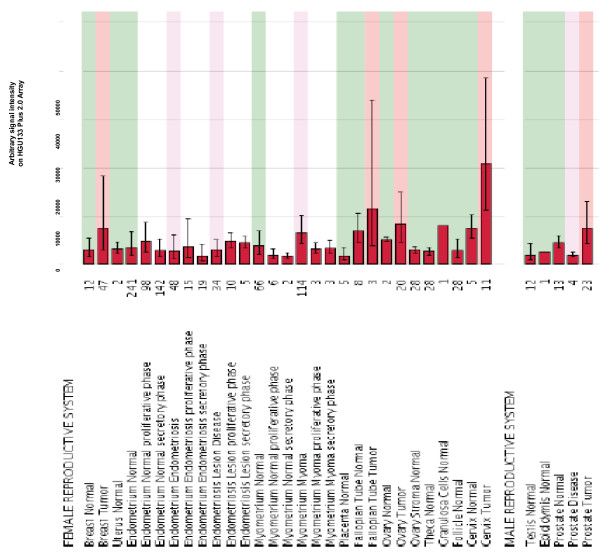
**Expression of SRD5A1 in the female and male reproductive tract**. SRD5A1 over-expression was observed in breast, fallopian tube, ovary, cervix and prostate cancer on the Affymetrix HGU133Plus2.0 array with the SRD5A1-specific probeset 204675_at.

The expression of SRD5A1 in additional tumors was determined by Array Northern. Besides in NSCLC, over-expression of SRD5A1 was observed in breast cancer, and also in ovary, cervix and prostate cancer. In the Array Northern shown in Figure 3, the gene expression values for SRD5A1-based on the Affymetrix HGU133Plus2.0 array data-are displayed as a bar graph of the geometric mean values of the expression value on an arbitrary scale. The expression pattern of SRD5A1 was also analyzed in a large panel of normal human tissues. Strong expression signals were detected in skin, brain, liver, esophagus and small intestine (Additional File 4A). Strong expression in the skin has previously been noted [9,23]. Expression of SRD5A1 was also detected in many different human cell lines on the Array Northern (Affymetrix HGU133Plus2.0 array), with highest levels observed in breast cancer cells (Additional File 4B).

### SRD5A1 knock-down efficiency at the mRNA level

**Figure 4:**
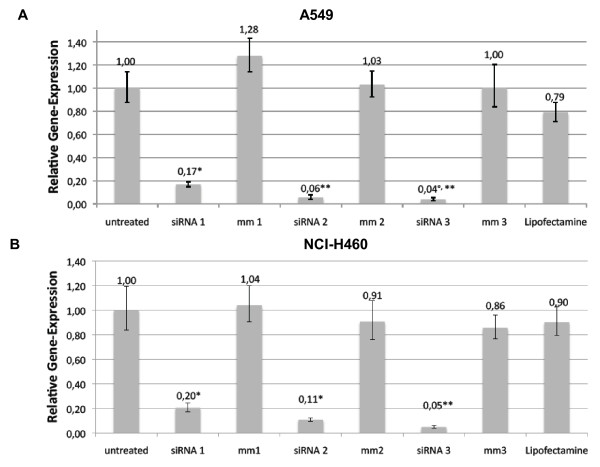
**Relative gene expression of SRD5A1 normalized to cyclophilin after 24 h of siRNA treatment in A549 (A) and NCI-H460 (B) cells**. Experiments were performed in triplicate with 10 pmol siRNA and 5 μl Lipofectamine™ 2000. (A) A549: * significant difference to untreated (siRNA 1: P = 0.001), **significantly smaller than 5% (siRNA 2: P < 0.001, siRNA 3: P < 0.001). (B) NCI-H460: *significant difference to untreated (siRNA 1: P = 0.2, siRNA 2: P = 0.11), **significantly smaller than 5% (siRNA 3: P = 0.05).

NSCLC is the most frequent lung cancer type (approximately 80% of cases) and the focus of the present study. SRD5A1 expression was very similar in the four NSCLC cell lines analyzed (Figure 2C) and A549 and NCI-H460 cells were chosen for further analyses. Starting with 50 ng of cDNA for analysis with qRT-PCR, the C_t _was approximately 26 for SRD5A1 in both cell lines compared to 18 for cyclophilin A, an abundant housekeeping mRNA. The ΔCT value for both cell lines was approximately 8 cycles. To establish the lowest amount of siRNAs still inducing an effective knock-down, concentrations ranging from 10 pmol to 100 pmol of siRNA per well of a six-well culture dish were used and the knock-down was measured at 24 h, 48 h, and 72 h post-transfection. The lowest siRNA concentration causing an efficient knock-down was used for the subsequent experiments in order to minimize potential off-target effects [24],[25]. In these experiments, 10 pmol siRNA transfected with 5 μl of Lipofectamine™ 2000 were sufficient to achieve a very effective reduction, down to less than 10% in comparison to the untreated control in both A549 and NCI-H460 cells (Additional File 5). "siRNA 1-3" refers to the target siRNAs 1, 2 and 3 in all diagrams while "mm 1-3" refers to the mismatch controls 1, 2 and 3. The knock-down of SRD5A1 was stable at 48 h and 72 h in both cell lines, and higher amounts of siRNA (50 pmol and 100 pmol) did not further reduce SRD5A1 levels (data not shown). Due to the toxicity observed in the proliferation assays, the knock-down assays were conducted again with a reduced amount of Lipofectamine™ 2000, with 2.5 μl being used per well. The results are displayed in Figure 4. Here also, very good knock-down efficiency was achieved and the effects were stable over 72 h (data not shown).

### SRD5A1 knock-down efficiency at the protein level

**Figure 5:**
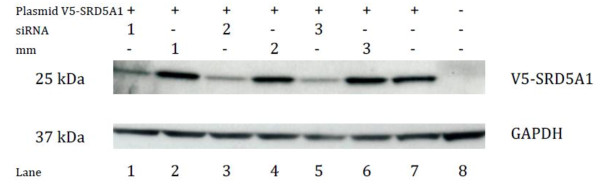
**Immunoblot after over-expression of SRD5A1-V5 in A549 cells and subsequent siRNA transfection**. Lysates were prepared 24 h after siRNA transfection, The percentage signal intensity compared to lane 7 is given in brackets. The 25 kDa band corresponds to SRD5A1-V5, the 37 kDa band to GAPDH. Lane 1: siRNA 1 (31%); lane 2: mm 1 (104%); lane 3: siRNA 2 (22%); lane 4: mm 2 (87%); lane 5: siRNA 3 (29%); lane 6: mm 3 (107%); lane 7: plasmid (w/o subsequent siRNA treatment) (100%); lane 8: untreated (0%).

Following establishment of the knock-down conditions at the RNA level, an immunoblot was performed to analyze the reduction of protein levels. The two anti-SRD5A1 antibodies used did either not allow to detect bands or showed multiple non-specific bands on the immunoblot of A549 or NCI-H460 cells (not shown). Similar results were obtained in cells transfected with an SRD5A1 expression plasmid (not shown). Thus these antibodies could not be used for evaluating the SRD5A1 protein levels after siRNA-mediated knock-down. V5-tagged SRD5A1 protein over-expressed in A549 or NCI-H460 was, however, detected as a specific band with an anti-V5-antibody. This antibody was subsequently used to analyze whether siRNAs directed against human SRD5A1 induced knock-down of SRD5A1 at the protein level. The results are displayed in Figure 5 for the A549 cells. They show that the SRD5A1-V5 protein levels were decreased by 69-78% 24 h after siRNA transfection in comparison to the mismatch controls. A reduction of SRD5A1-V5 levels was still detected at 48 h, although the levels of transfected SRD5A1-V5 were already declining at this time point (data not shown). Similar observations were made to a lesser extent in NCI-H460 cells (not shown). The results indicate that the three siRNAs against SRD5A1 reduced the protein expression of SRD5A1-V5 after 24 h by 69-78% in A549 and by 20-49% in NCI-H460, suggesting a half-life of approximately 12 h in A549 cells and 20-30 h in NCI-H460 cells.

### Proliferation assay

**Figure 6:**
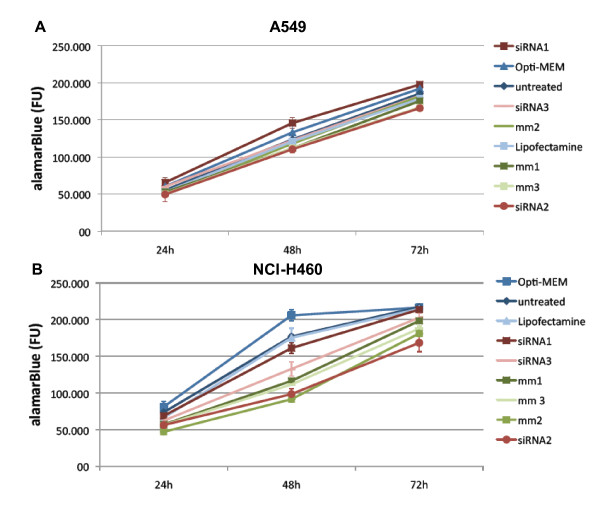
**Proliferation assay after siRNA treatment of A549 (A) and NCI-H460 (B) cells**. Experiments were conducted in triplicate with 10 pmol siRNA and 2.5 μl Lipofectamine™ 2000. No significant differences were observed between target siRNA-treated and mismatch siRNA-treated cells in either cell line.

The lung cancer cell lines were grown in medium supplemented with FCS, which contains 0.03 ng/ml testosterone. Next, A549 or NCI-H460 cells were transfected with 10 pmol of each siRNA in presence of 2.5 μl Lipofectamine™ 2000. An Alamar Blue proliferation assay was then performed (Figure 6). In comparison to the preceding proliferation assay with 5 μl Lipofectamine™ 2000 (Additional File 6), only little toxicity was observed. No noticeable difference was visible in the proliferation between cells with silenced SRD5A1 and the mismatch controls.

### Cell cycle analysis

**Figure 7:**
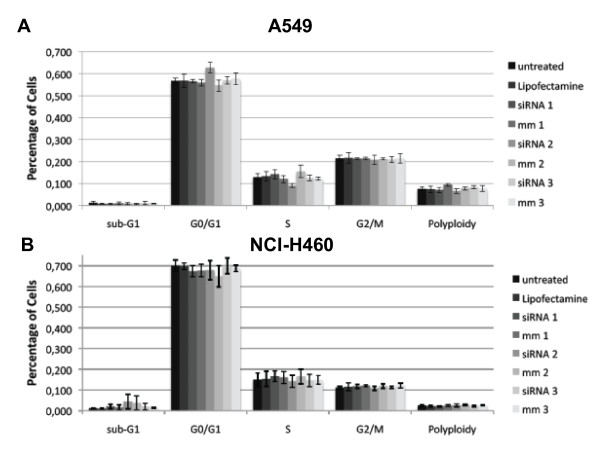
**Cell cycle analysis 72 h after siRNA treatment of A549 (A) and NCI-H460 (B) cells**. Experiments were conducted in triplicate with 10 pmol siRNA and 2.5 μl Lipofectamine™ 2000. The cell distribution at 24 h and 48 h did not vary from the results at 72 h and is not shown.

In addition to the proliferation assays, flow cytometry was performed to analyze cell cycle distribution and apoptosis/necrosis assays to find out whether more discrete changes were noticeable after the silencing of SRD5A1 expression. The results of the cell cycle analysis 72 h after knock-down with 2.5 μl Lipofectamine™ 2000 and 10 pmol siRNA are displayed in Figure 7. The sub-G1 peak represents apoptotic cells because of the enzymatic digestion of DNA in the process of apoptosis. Polyploidy on the other hand refers to cells with a hyperdiploid chromosome set. Knock-down of SRD5A1 in A549 or NCI-H460 cells did not alter the percentage of cells in the different phases of the cell cycle, nor the number of cells with sub-G1 DNA content or of aneuploid cells. The distribution of cells at 24 h and 48 h did not vary from the results at 72 h and the data are therefore not shown.

### Apoptosis/necrosis assay

**Figure 8:**
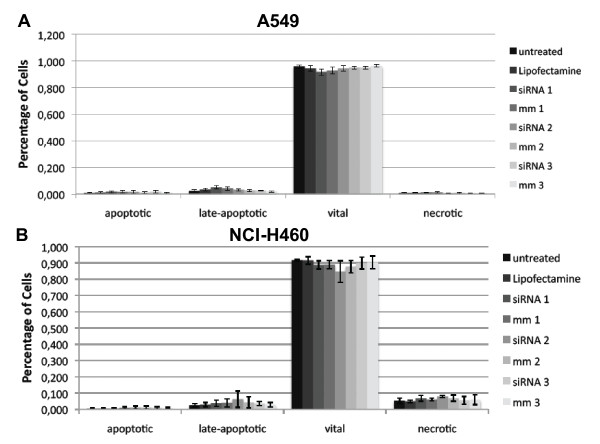
**Apoptosis/necrosis assay 72 h after siRNA treament of A549 (A) and NCI-H460 (B) cells**. Experiments were conducted in triplicate with 10 pmol siRNA and 2.5 μl Lipofectamine™ 2000. The cell distribution at 24 h and 48 h did not vary from the results at 72 h and is not shown.

The results of the apoptosis/necrosis assay 72 h after knock-down of SRD5A1 in A549 or NCI-H460 cells with 2.5 μl Lipofectamine™ 2000 and 10 pmol siRNA are displayed in Figure 8. There were no observable differences in cell viability between treatment and control groups in both cancer cell lines. The distribution of cells at 24 h and 48 h did not vary from the results at 72 h and the data are therefore not shown.

### Chemosensitivity assay

**Figure 9:**
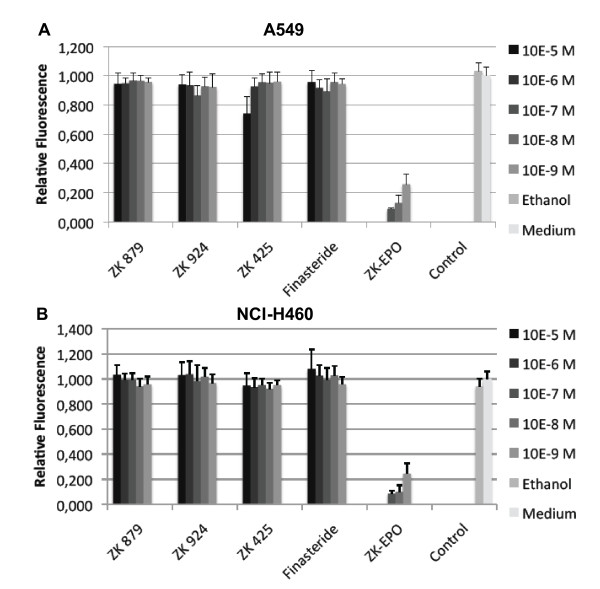
**Chemosensitivity assay 120 h post-treatment in A549 (A) and NCI-H460 (B) cells**. (A) A549: Sagopilone (ZK-EPO)-treated groups showed significantly less cell viability at each concentration than the vehicle control (P < 0.001). The ZK 425 group treated at 10^-5 ^M showed significantly less cell viability than the vehicle control (P = 0.019). (B) NCI-H460: Sagopilone-treated groups showed significantly less cell viability at each concentration than the vehicle control (P < 0.001).

Three selective inhibitors of SRD5A1 (ZK 879, ZK 924 and ZK 425 [20], all belonging to the 17-methylene-4-azasteroids family [19]) and Finasteride, a specific SRD5A2 inhibitor, were used for chemosensitivity assays in order to determine whether inhibition of the enzymatic activity of SRD5A1 had an effect on cell proliferation. The results obtained after 120 h of treatment are presented in Figure 9. Photometrical measurement at 0 h showed equal cell amounts to be present in all treatment and control groups. At 72 h, only Sagopilone (ZK-EPO)-treated cells showed a decrease in cell viability. The effect was even more pronounced after 120 h where a dramatic effect on cell viability was observed. Conversely, Finasteride and the SRD5A1 inhibitors, except ZK 425 at the highest concentration used (10^-5 ^M), had no effect on cell proliferation.

## Discussion

Dysregulated expression of discrete sets of genes is frequently observed in many tumor types, including lung cancer [26-28]. In case an over-expressed gene is causally involved in cancer growth, its specific blockade may show clinical benefit. This is preferentially achieved by small molecule or antibody approaches. More recently, targets with little druggability have been addressed by RNA interference in order to reduce expression levels, and first clinical successes have been reported [29,30].

Elevated levels of SRD5A1 and SRD5A2 have been reported in prostate cancer and a correlation with the severity of the disease linked to increased dihydrotestosterone levels was documented [14]. SRD5A1 and SRD5A2 are also consistently over-expressed in breast cancer, which increases the levels of progesterone metabolites possibly involved in cell proliferation [13]. Indeed, we found SRD5A1 to be markedly elevated in prostate and breast cancer, and also in several breast cancer cell lines.

The main finding of the present study is that SRD5A1 is significantly over-expressed in NSCLC. This was observed in clinical samples originating from two different patient populations which were analyzed either by microarray hybridization or by qRT-PCR. High expression of SRD5A1 expression was furthermore measured in the lung cancer cell lines A549 and NCI-H460. A study was therefore initiated to determine whether SRD5A1 played a determinant role in lung cancer cell proliferation. For that, conditions for an efficient silencing of SRD5A1 expression in these two cell lines via RNA interference were first established. Proliferation assays and flow cytometry analyses were then conducted. In addition, three SRD5A1 inhibitors were used for chemosensitivity assays in order to determine if any anti-proliferative effects could be observed.

Knock-down of SRD5A1 was conducted with the lowest amount of siRNA still providing an effective reduction of expression levels [25]. With as little as 10 pmol siRNA per well of a six-well culture dish, a very good knock-down to under 20% of SRD5A1 mRNA in all cases and under 10% of SRD5A1 mRNA in most cases, when compared to the untreated control, was achieved. Higher amounts of siRNA (50 and 100 pmol) did not result in a more effective knock-down. The concentration of 10 pmol per well was therefore used for all following experiments.

Beside the knock-down of target gene expression, reduction of the target protein levels needs additionally to be proven [31]. Despite an effective knock-down of mRNA, the target protein can persist in the cells, for example due to a long half-life of the protein. The half-life of SRD5A1 has been calculated to be 20-30 h in CHO cells by Russell and Wilson [23]. Due to non-specific binding, the two commercially available SRD5A1 antibodies could not be used to quantify SRD5A1 protein levels after siRNA treatment. To determine in an indirect way whether the three selected siRNAs indeed decreased the SRD5A1 protein content of the cells, over-expression of SRD5A1-V5 was conducted together with siRNA transfection. In A549 cells, a knock-down was clearly observed after 24 h, the estimated half-life of SRD5A1-V5 being approximately 12 h, and thus significantly shorter than what was observed previously in CHO cells. Knock-down of over-expressed SRD5A1-V5 at the protein level was also achieved in NCI-H460 lung cancer cells, the estimated half-life being approximately 20-30 h.

After establishing the optimal conditions for knock-down of SRD5A1, functional assays were conducted. In proliferation assays using AlamarBlue, no differences between target siRNA-treated groups and control groups were observed. Flow cytometry was performed to check for more subtle effects. Differences between the different treatment groups could neither be identified in cell cycle distribution nor in the apoptosis/necrosis assay. In the chemosensitivity assays, the only observable inhibition of proliferation occurred in the group treated with 10^-5 ^M ZK425 in A549 cells. As the other concentrations did not result in any inhibition of proliferation, this is likely to represent a non-specific effect linked to the very high concentration of compound used. In view of these results, it is unlikely that SRD5A1 contributes significantly to the proliferation of NSCLC. The conduction of similar experiments in cell lines originating from lung SCC is planned (e.g. in NCI-H520 or SW-900 cells), since an up-regulation of SRD5A1 has been observed in tissue samples of SCC as well. Before that, up-regulation of SRD5A1 needs to be confirmed in candidate cell lines.

On the other hand, it cannot be excluded that the conditions used here did not allow the proper assessment of the role of SRD5A1 in lung cancer. Cultivating cells in monolayers alters their requirements for growth factors and stimulatory agents, and gene expression profiling studies show significant differences in comparison to cells cultivated in three-dimensional conditions (e.g. spheroid-forming cells), which may better reflect the situation in the tumor [32,33]. Also, the signals originating from neighboring stromal cells and from the extra-cellular matrix influence the pathways that are essential for tumor cell growth [34,35]. In view of the availability of selective SRD5A1 inhibitors, one might consider *in vivo *testing in nude mice xenografted with human lung tumor models as a more sophisticated approach to address these points.

## Conclusions

SRD5A1 was found to be up-regulated in NSCLC by microarray analysis and qRT-PCR. To elucidate whether SRD5A1 levels influenced the proliferation of NSCLC cells, knock-down experiments with specific siRNAs were conducted in A549 and NCI-H460 lung cancer cells. Despite efficient knock-down, no changes in proliferation, cell cycle distribution or apoptosis/necrosis were observed. Moreover, blockade of the enzymatic activity of SRD5A1 with three specific inhibitors did not reduce proliferation of A549 and NCI-H460 cells. In summary, SRD5A1 knock-down or inhibition does not affect proliferation of the NSCLC cell lines analyzed.

## List of abbreviations

C_t_: threshold cycle; FCS, fetal calf serum; NAT: normal adjacent tissue; NSCLC: non-small cell lung cancer; PCA: principal component analysis; qRT-PCR: quantitative real-time PCR; SCC: squamous cell carcinoma; SRD5A1: 5-alpha-reductase type I; SRD5A2: 5-alpha-reductase type II; SRD5A3: 5-alpha-reductase type III.

## Competing interests

FK received a scholarship from Bayer HealthCare during the conduction of the experiments for his doctoral thesis. BH and AS are employees of Bayer HealthCare. No conflict of interest was encountered.

## Authors' contributions

FK conducted the experiments on A549 and NCI-H460 cells. AS performed Affymetrix microarray experiments and analyzed SRD5A1 and SRD5A2 in the Array Northern database. TK and GD participated in the experimental design and contributed to the statistical analysis of the data. BH designed, coordinated and supervised the experimental studies. All authors read and approved the final manuscript.

## Supplementary Material

Additional File 1 **Location of the siRNA 1-3 binding sites in the human SRD5A1 cDNA sequence**. The position of the siRNA 1-3 binding sites in the human SRD5A1 sequence is shown. Letters with green background indicate non-coding regions of the human SRD5A1 sequence (Gene ID: 6715; RefSeq Seq: NM_001047.2), letters with white background represent the coding sequence. colored letters indicate the binding site of the specific siRNAs (blue: siRNA 1, green: siRNA 2, red: siRNA 3). Click here for file

Additional File 2 **Expression construct for CMV-driven expression of human V5-tagged SRD5A1 protein**. A map of the vector used for expression of human SRD5A1 is shown. The SRD5A1 cDNA was cloned into the mammalian expression vector pcDNA/V5-His (Invitrogen) upstream of the V5 tag in order to generate a SRD5A1 fusion protein with the V5 tag at the C-terminus. Click here for file

Additional File 3 **Structure of 17-methylene-4-azasteroids**. The general chemical structure formula of 17-methylene-4-azasteroids is shown [20]. For a more detailed structure, also see the review by Aggarwal et al. [19]. Click here for file

Additional File 4 **SRD5A1 expression in normal tissues and in cell lines**. The expression pattern of SRD5A1 was analyzed in a panel of human tissues (A) and cell lines (B) in the Array Northern database. Probeset 204675_at which interrogates SRD5A1 on the Affymetrix HGU133Plus2.0 array is shown. On the x-axis the human tissues and cell lines are shown sorted by type. The number of replicates analyzed is indicated. The y-axis depicts arbitrary expression units. In normal tissues, the highest transcript levels were detected in skin, esophagus, liver, small intestine, and in neuronal tissue. A relatively weak expression was observed in normal lung tissue. The cell lines with highest SRD5A1 expression are derived from breast cancer. Click here for file

Additional File 5 **Relative gene expression of SRD5A1 normalized to cyclophilin 24 h after siRNA-mediated silencing in A549 (A) and NCI-H460 (B) cells**. SRD5A1 silencing experiments were performed in A549 and NCI-H460 cells in triplicate with 10 pmol siRNA and 5 μl Lipofectamine™ 2000. (A) A549: *significant difference to untreated (mm 1: P = 0.001)¸ **significantly smaller than 5% (siRNA 1: P = 0.001, siRNA 2: P < 0.001, siRNA 3: P < 0.001). (B) NCI-H460: *significant difference to untreated (siRNA 1: P = 0.010, siRNA 2: P = 0.004, siRNA 3: P < 0.001). Click here for file

Additional File 6 **Proliferation assay after siRNA treatment of A549 (A) and NCI-H460 (B) cells**. Proliferation experiments were conducted in triplicate with 10 pmol siRNA and 5 μl Lipofectamine™ 2000. Significantly reduced proliferation was observed in all Lipofectamine™ 2000-treated groups (P < 0.001-P = 0.04) in both cell lines. Click here for file
