# Supplementary figures and images for: 5-alpha-reductase type I (SRD5A1) is up-regulated in non-small cell lung cancer but does not impact proliferation, cell cycle distribution or apoptosis
Source: Cancer Cell Int. 2012 Jan 18;12:1. doi: 10.1186/1475-2867-12-1 (PMC3269976; doi:10.1186/1475-2867-12-1)

## Slide 1
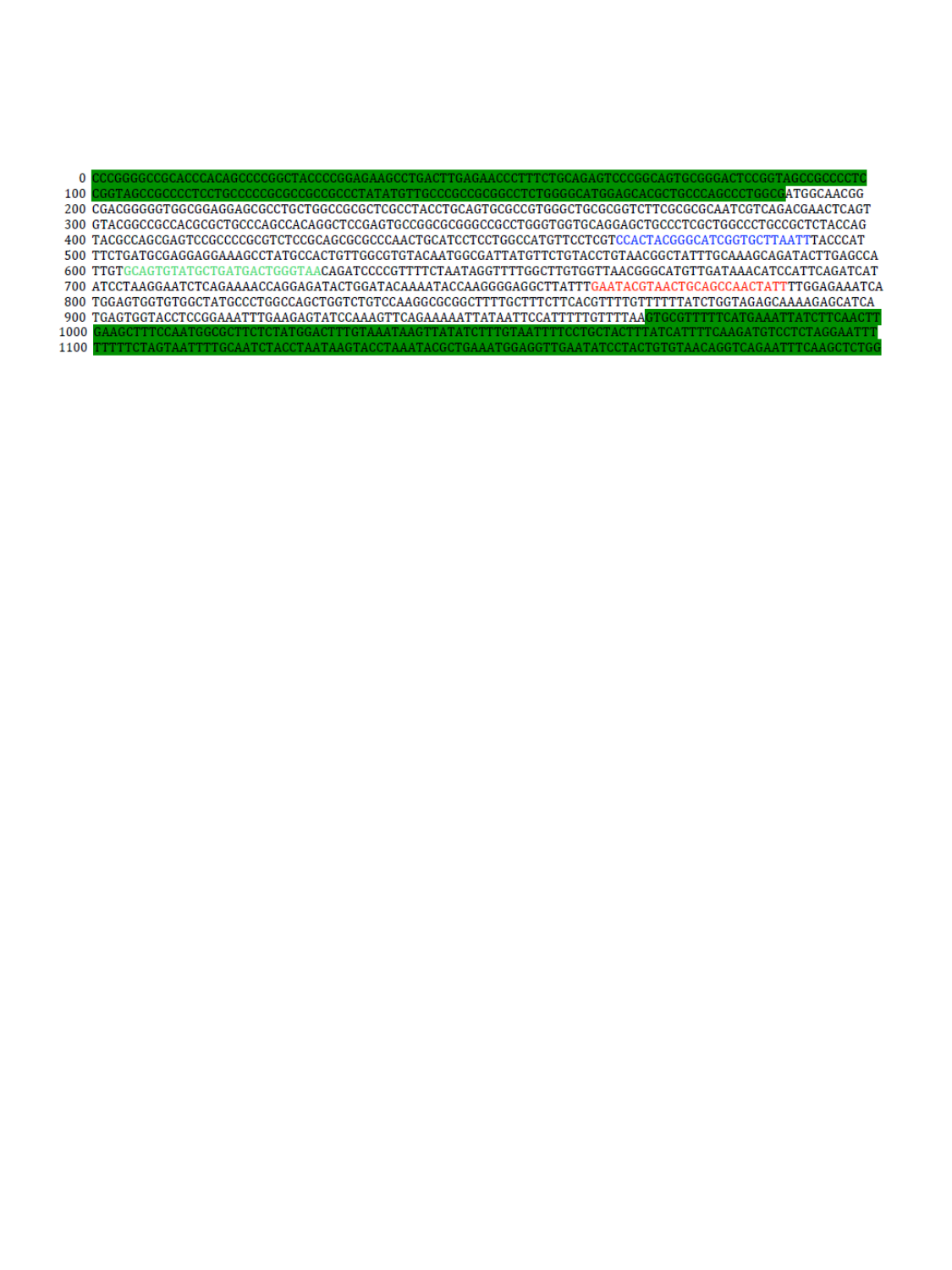

Supplement: Additional File 1 — Location of the siRNA 1-3 binding sites in the human SRD5A1 cDNA sequence. The position of the siRNA 1-3 binding sites in the human SRD5A1 sequence is shown. Letters with green background indicate non-coding regions of the human SRD5A1 sequence (Gene ID: 6715; RefSeq Seq: NM_001047.2), letters with white background represent the coding sequence. colored letters indicate the binding site of the specific siRNAs (blue: siRNA 1, green: siRNA 2, red: siRNA 3). [file 1475-2867-12-1-S1.PPT]

## Slide 1
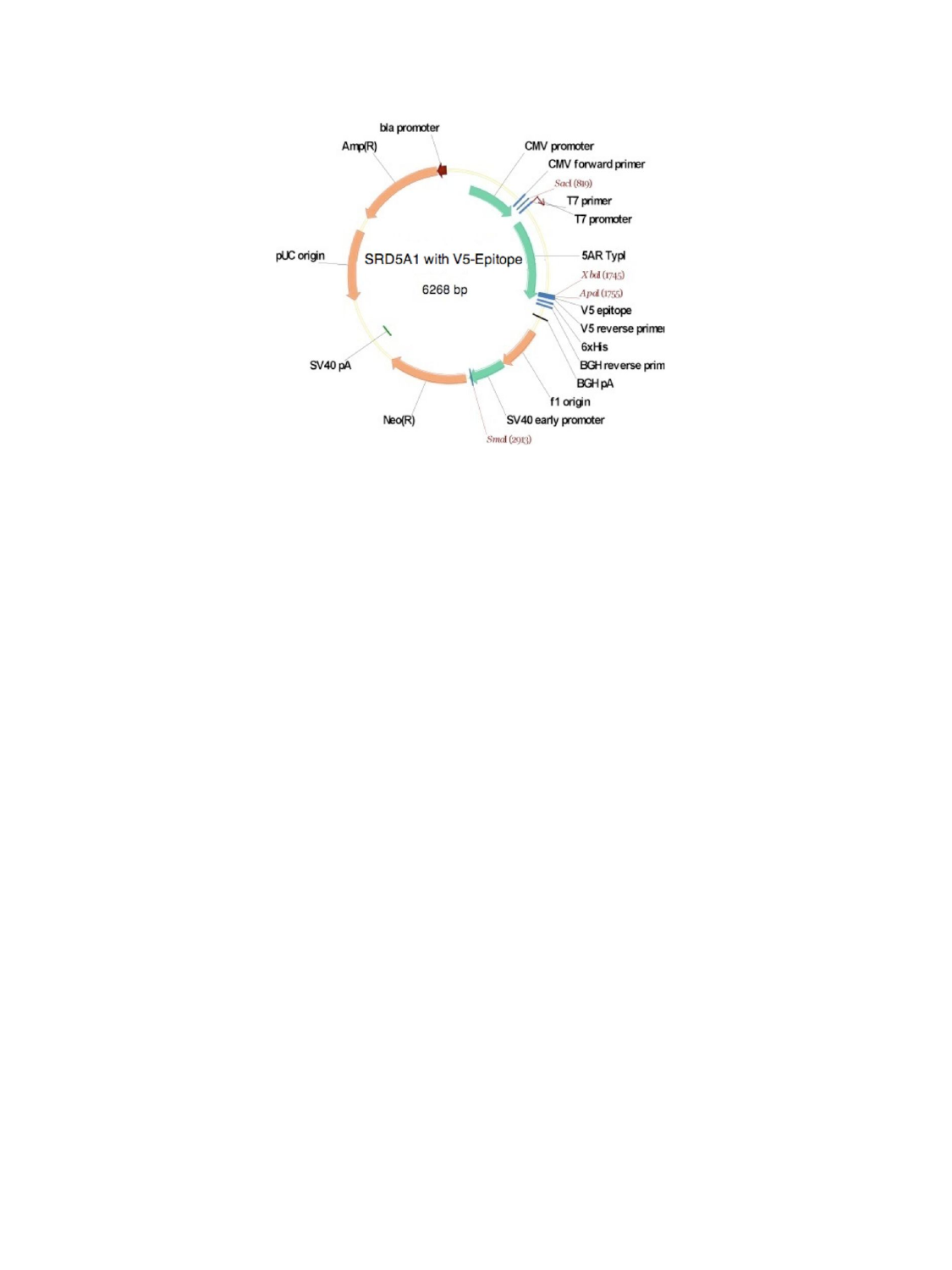

Supplement: Additional File 2 — Expression construct for CMV-driven expression of human V5-tagged SRD5A1 protein. A map of the vector used for expression of human SRD5A1 is shown. The SRD5A1 cDNA was cloned into the mammalian expression vector pcDNA/V5-His (Invitrogen) upstream of the V5 tag in order to generate a SRD5A1 fusion protein with the V5 tag at the C-terminus. [file 1475-2867-12-1-S2.PPT]

## Slide 1
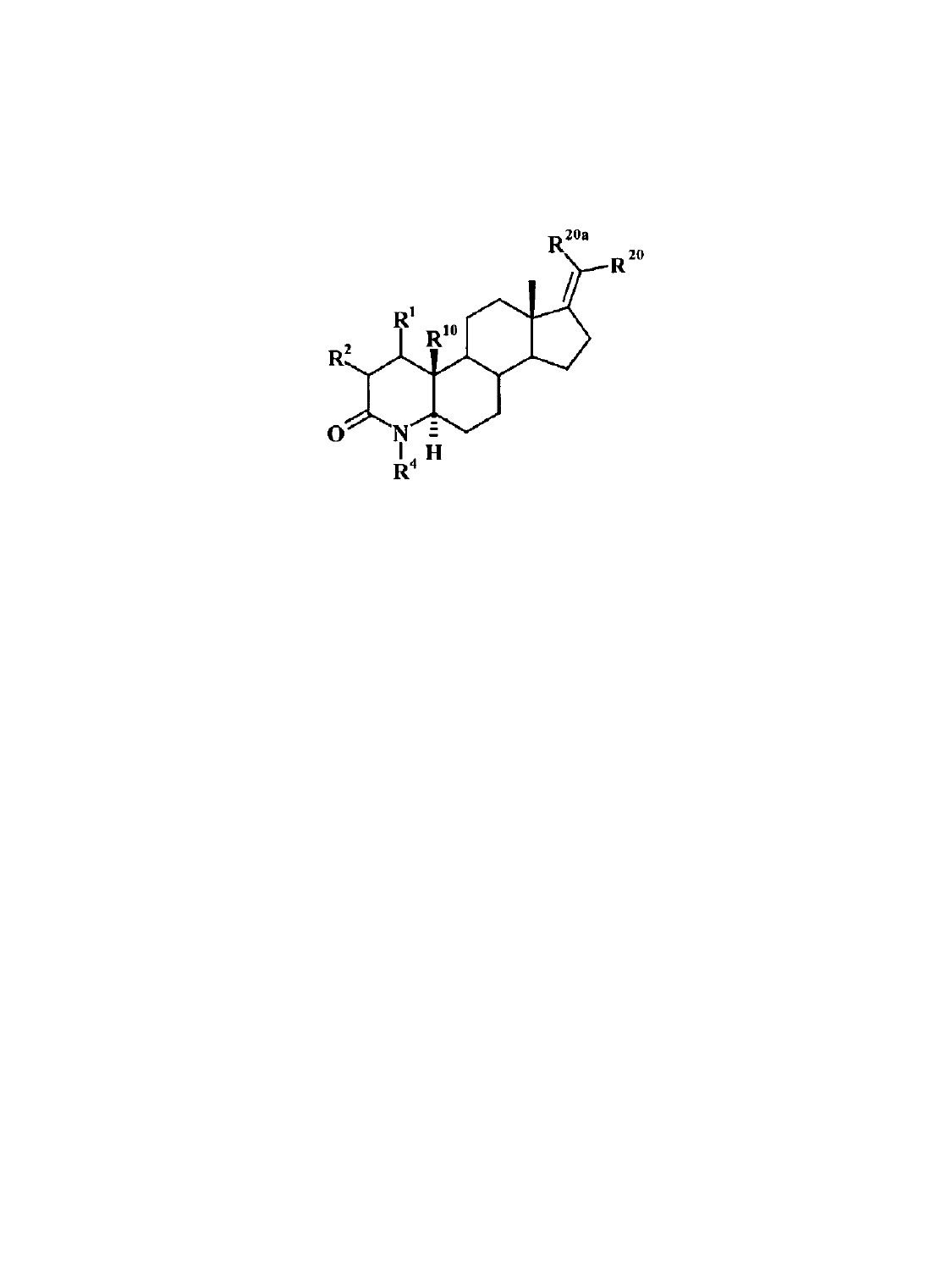

Supplement: Additional File 3 — Structure of 17-methylene-4-azasteroids. The general chemical structure formula of 17-methylene-4-azasteroids is shown [20]. For a more detailed structure, also see the review by Aggarwal et al. [19]. [file 1475-2867-12-1-S3.PPT]

## Slide 1
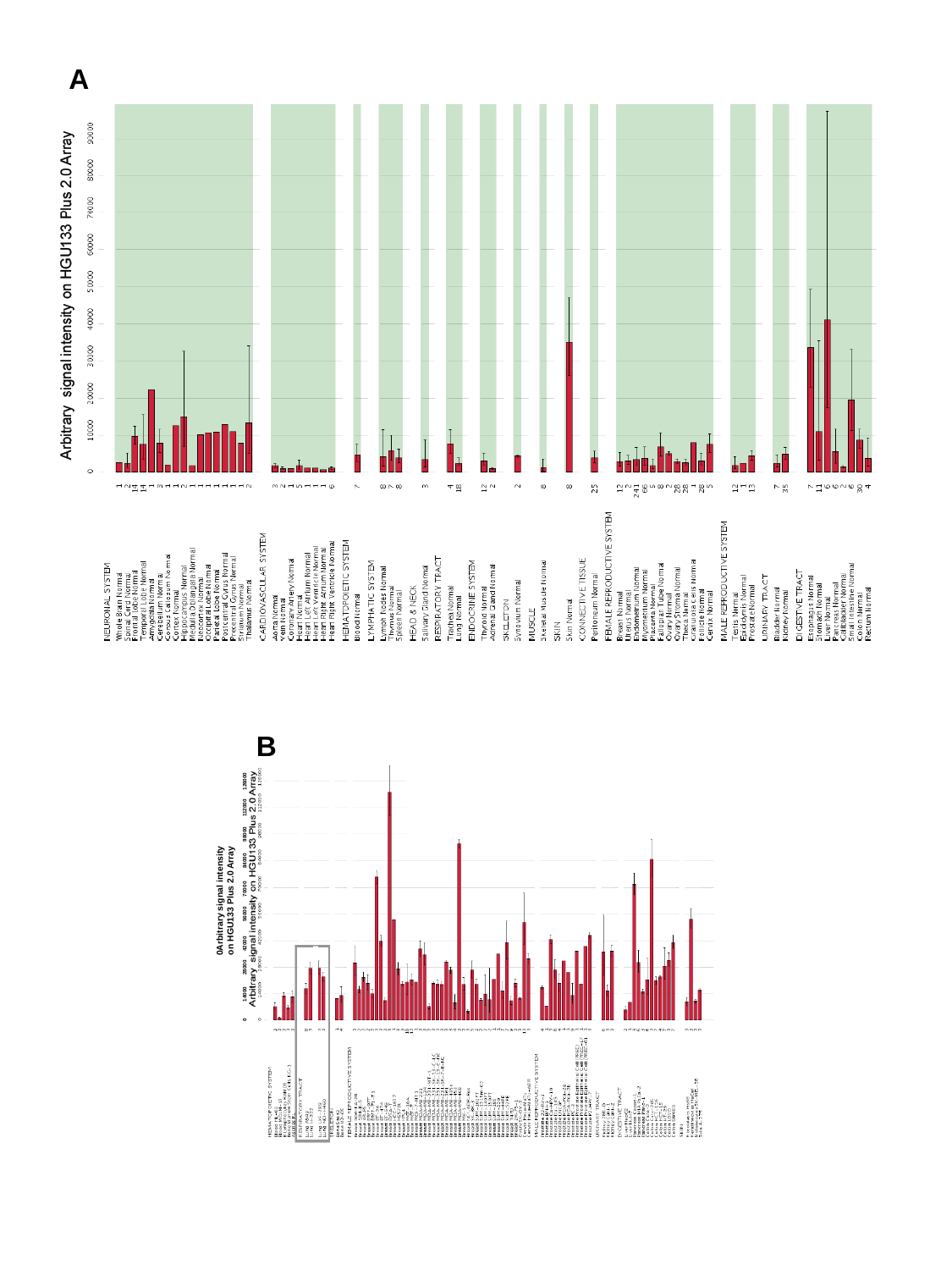

A
B
0Arbitrary signal intensity
on HGU133 Plus 2.0 Array
0 14000 28000 42000 56000 70000 84000 98000 112000 126000

Supplement: Additional File 4 — SRD5A1 expression in normal tissues and in cell lines. The expression pattern of SRD5A1 was analyzed in a panel of human tissues (A) and cell lines (B) in the Array Northern database. Probeset 204675_at which interrogates SRD5A1 on the Affymetrix HGU133Plus2.0 array is shown. On the x-axis the human tissues and cell lines are shown sorted by type. The number of replicates analyzed is indicated. The y-axis depicts arbitrary expression units. In normal tissues, the highest transcript levels were detected in skin, esophagus, liver, small intestine, and in neuronal tissue. A relatively weak expression was observed in normal lung tissue. The cell lines with highest SRD5A1 expression are derived from breast cancer. [file 1475-2867-12-1-S4.PPT]

## Slide 1
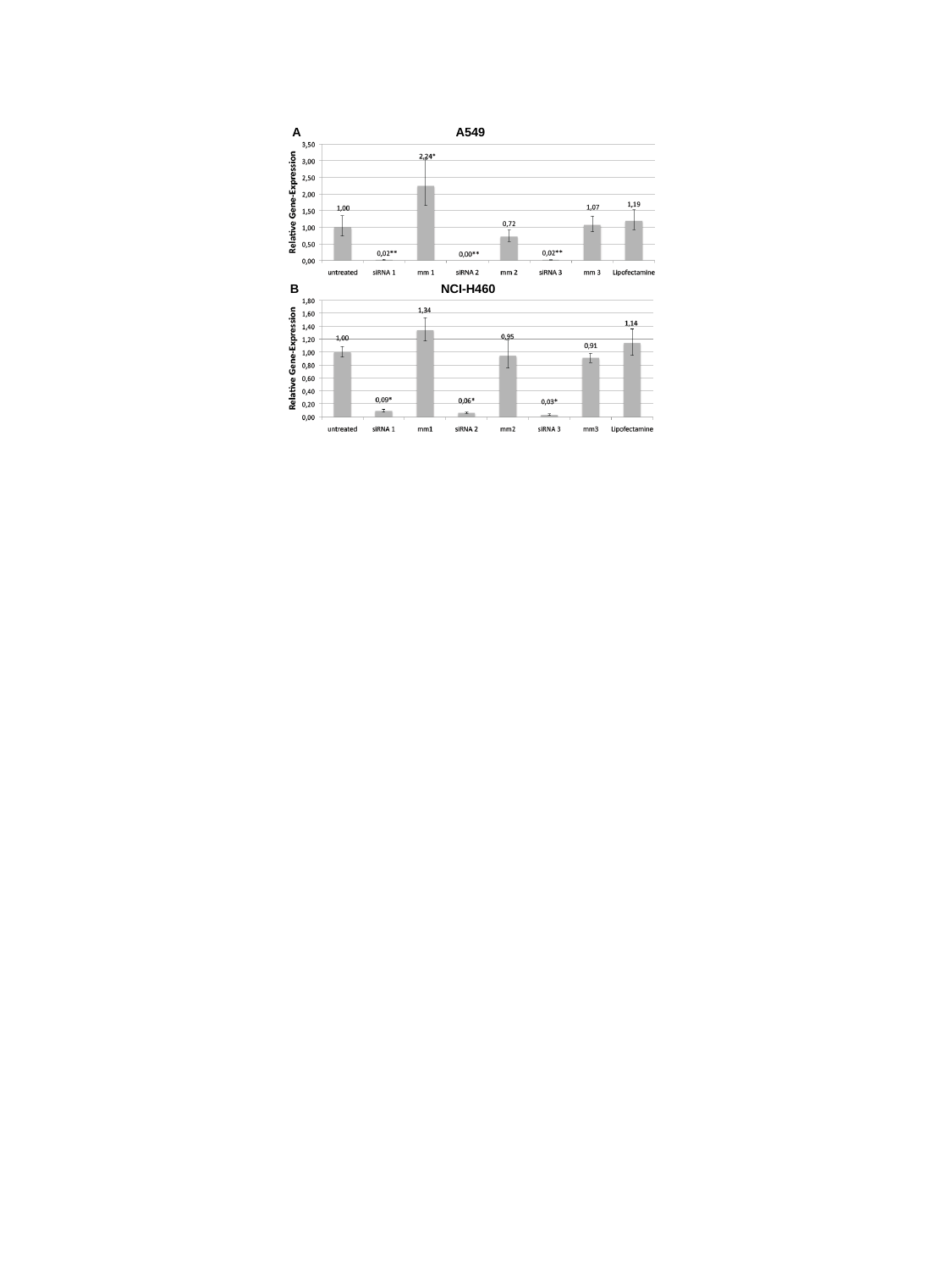

A A549
B NCI-H460

Supplement: Additional File 5 — Relative gene expression of SRD5A1 normalized to cyclophilin 24 h after siRNA-mediated silencing in A549 (A) and NCI-H460 (B) cells. SRD5A1 silencing experiments were performed in A549 and NCI-H460 cells in triplicate with 10 pmol siRNA and 5 μl Lipofectamine™ 2000. (A) A549: *significant difference to untreated (mm 1: P = 0.001)¸ **significantly smaller than 5% (siRNA 1: P = 0.001, siRNA 2: P < 0.001, siRNA 3: P < 0.001). (B) NCI-H460: *significant difference to untreated (siRNA 1: P = 0.010, siRNA 2: P = 0.004, siRNA 3: P < 0.001). [file 1475-2867-12-1-S5.PPT]

## Slide 1
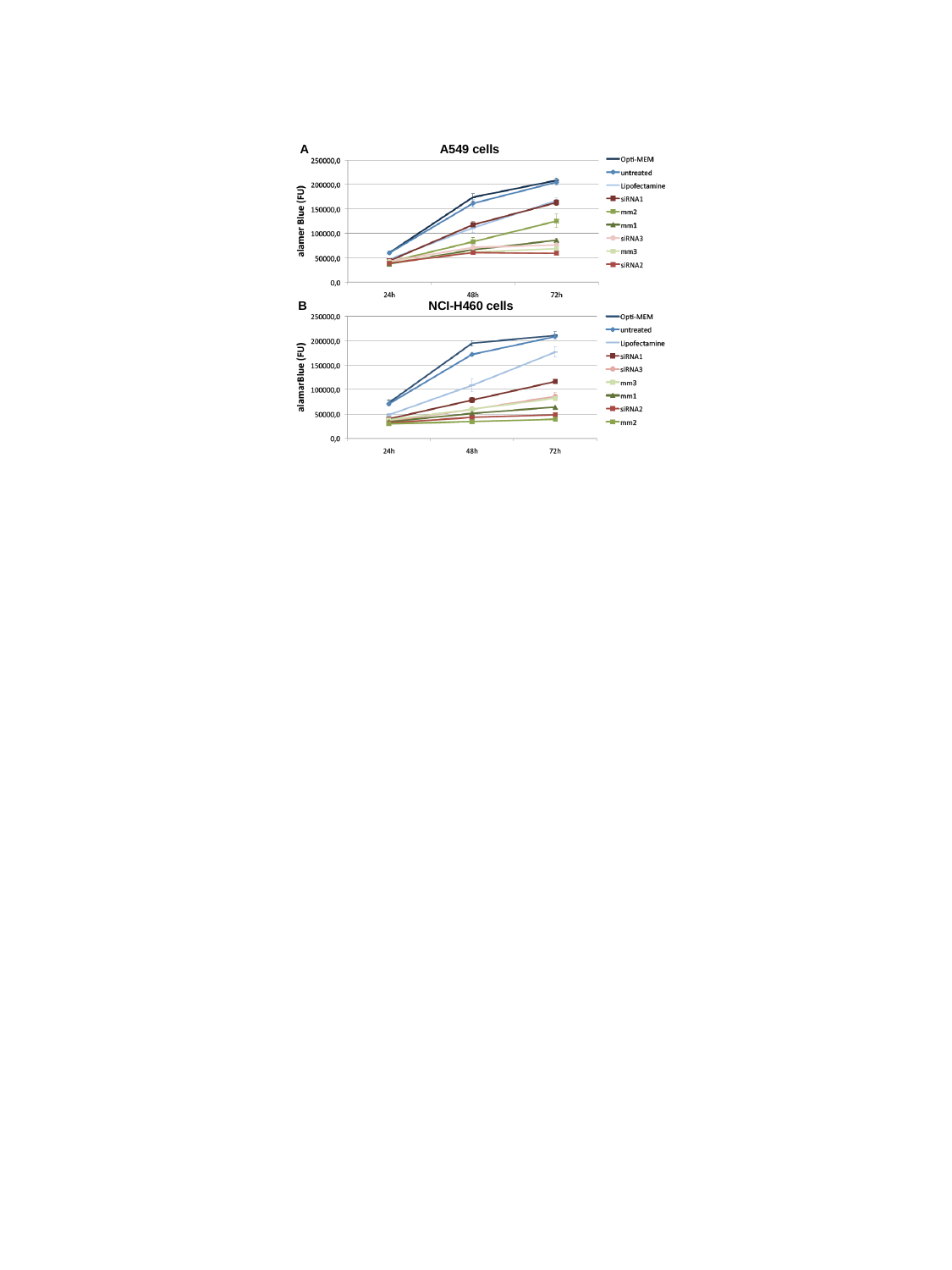

A A549 cells
B NCI-H460 cells

Supplement: Additional File 6 — Proliferation assay after siRNA treatment of A549 (A) and NCI-H460 (B) cells. Proliferation experiments were conducted in triplicate with 10 pmol siRNA and 5 μl Lipofectamine™ 2000. Significantly reduced proliferation was observed in all Lipofectamine™ 2000-treated groups (P < 0.001-P = 0.04) in both cell lines. [file 1475-2867-12-1-S6.PPT]
